# Nanoparticle-based multivalent human antibodies offer potent and broad neutralization against Omicron sublineages

**DOI:** 10.1038/s41392-023-01512-6

**Published:** 2023-08-02

**Authors:** Yizhen Wang, Feibo Song, Siling Wang, Miaolin Lan, Tingdong Li, Huilin Guo, Yali Zhang, Shengxiang Ge, Zizheng Zheng, Ningshao Xia

**Affiliations:** 1 State Key Laboratory of Molecular Vaccinology and Molecular Diagnostics; National Institute of Diagnostics and Vaccine Development in Infectious Diseases, School of Public Health, School of Life Sciences, Xiamen University, Xiamen, China; 2 Xiang An Biomedicine Laboratory, Xiamen, China; 3 Research Unit of Frontier Technology of Structural Vaccinology of Chinese Academy of Medical Sciences, Xiamen, China

**Keywords:** Infectious diseases, Nanobiotechnology

**Dear Editor**

As of January 2023, coronavirus disease 2019 (COVID-19) induced by severe acute respiratory syndrome coronavirus 2 (SARS-CoV-2) has caused more than 6.7 million deaths, with SARS-CoV-2 variants continuing to alter the trajectory of the COVID-19 pandemic. Indeed, the prevalence of the newly emergent Omicron sublineages, particularly BA.4/5 and XBB, has highlighted the critical need for the design and production of broadly potent neutralization antibodies to efficiently combat SARS-CoV-2 variants.

Neutralizing antibodies (nAbs) XMA01, XMA04, and XMA09, which target noncompeting antigenic sites in RBD (receptor binding domain), exhibit high neutralization potency against earlier disease variants (Supplementary Fig. 1a–d), as previously reported by us.^1^ However, these nAbs were incapable of providing sufficient neutralization potency against the Omicron sublineages (Supplementary Fig. 1e–g). Specifically, XMA01 showed a partially decreased (10- to 100-fold) neutralizing activity against BA.1, BA.1.1, BA.2, and BA.2.12.1, but was completely ineffective against sublineages BA.4/5 and XBB. We surmised that this is because residue F486, in forming several strong interactions, is the key epitope factor for XMA01.^1^ XMA04 also failed to neutralize the BA.4/5 sublineage, requiring IC_50_ values higher than 50,000 ng/mL to provide an effect. The broad nAb XMA09 showed moderately reduced (< 10-fold) neutralization against all Omicron sublineages compared with the prototype strain. These findings are consistent with recent studies^2^ and indicate the remarkable immune escape of Omicron sublineages. To circumvent this avoidance and re-establish the broad neutralization potencies of XMA01, XMA04, and XMA09, we used a self-assembling nanoparticle mi3 to multimerize nAbs as 60-valent neutralizers, and formed mi3 nanoparticle-based multivalent antibodies (XMA01-mi3, XMA04-mi3, and XMA09-mi3) through optimized SpyTag/SpyCatcher technology (Fig. 1a and Supplementary Fig. 2a–d).^3,4^ These neutralizers decorated with mi3 nanoparticles each showed good integrity and a stabilized structure (Fig. 1b, Supplementary Figs. 2e, f and 3a, b).

**Fig. 1:**
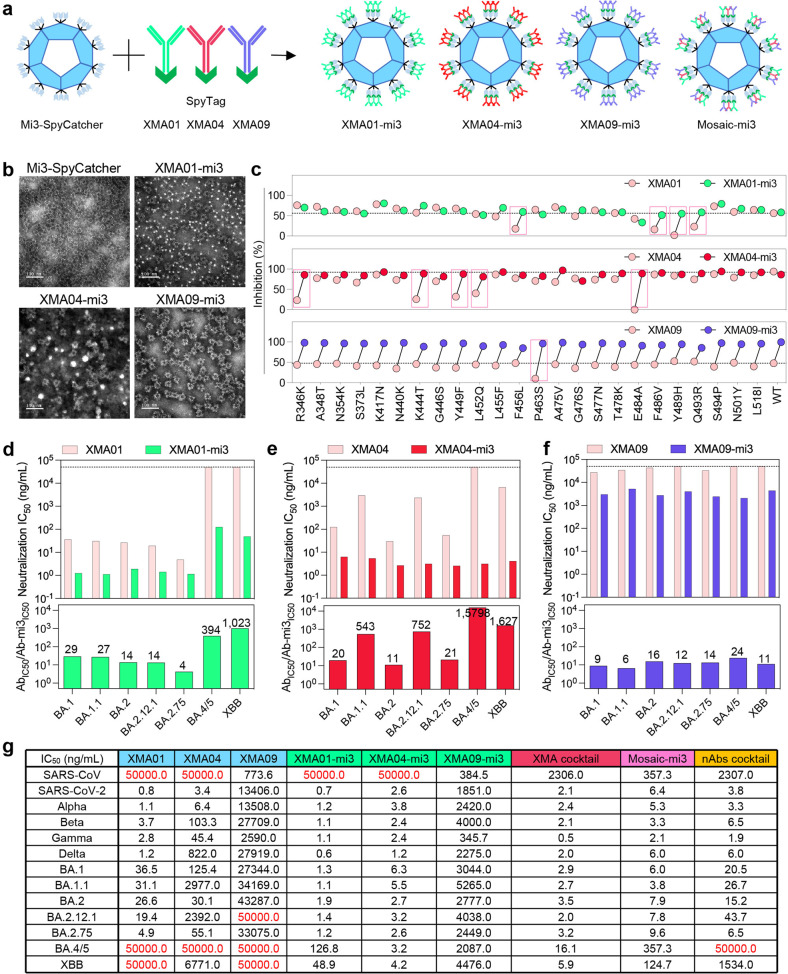
Rescuing the neutralization potency of antibodies against SARS-CoV-2 variants by nanoparticle-based multivalent transformation. **a** Schematic representation of nanoparticle-based multivalent transformation. **b** Negative stain electron micrographs of the mi3 and nanoparticle-based multivalent nAbs (mnAbs). (Scale bar 100 nm, representative of two independent experiments). **c** Resistance of nAbs and mnAbs to single-point mutations. Inhibition percentages for different neutralizers were determined against VSV pseudoviruses carrying the SARS-CoV-2 spike protein with single-residue substitutions. The experiment was repeated in duplicate. Data are expressed as means (*n* = 2). **d**–**f** Neutralization potency of XMA01/XMA01-mi3 **d**, XMA04/XMA04-mi3 **e**, and XMA09/XMA09-mi3 **f** against the Omicron sublineages. IC_50_ values were determined using LV pseudovirus with the SARS-CoV-2 spike protein. The experiments were repeated in triplicate. **g** A diagram showing the neutralization potencies of nAbs, mnAbs, the nAb cocktail (XMA01/XMA04/XMA09), the XMA cocktail (XMA01-mi3/XMA04-mi3/XMA09-mi3), and a Mosaic-mi3 with IC_50_ values against SARS-CoV, SARS-CoV-2 prototype strain, variants of concern (VOCs) and Omicron sublineages

As anticipated, we found that these nanoparticle-based multivalent neutralizing antibodies (hereafter, mnAbs) exhibited significantly stronger inhibitory activities against point-mutant pseudoviruses than the parental nAbs,^5^ indicating that the nanoparticle-based multivalent design provides an advantage for resistance to the escape mutations (Fig. 1c). Remarkably, the multivalent transformation of XMA09 improved its neutralizing activity against all mutants and the wild-type virus. Notably, XMA01-mi3 dramatically recovered the loss of neutralizing activity of XMA01 against the Omicron variant (IC_50_ values: ~1 ng/mL) (Supplementary Fig. 4a–c), and XMA04-mi3 showed potent and broad neutralization against all variants of concern (VOCs) (IC_50_ values: 1–10 ng/mL). Moreover, the neutralization potency of XMA04-mi3 against the Delta variant was 681-times improved as compared with its parental XMA04. XMA09-mi3 showed moderate increases in neutralization potency against VOCs. Next, we determined the improvements in the neutralization potencies of the nanoparticle-based multivalent XMA01-mi3, XMA04-mi3 and XMA09-mi3 antibodies against the Omicron sublineages, specifically BA.1.1, BA.2, BA.2.12.1, BA.2.75, BA.4/5 and XBB. As anticipated, the multivalent transformation recovered the neutralization losses observed with the parental antibodies, with neutralization efficacy against BA.4/5 (XMA01-mi3, XMA04-mi3 and XMA09-mi3 with IC_50_ values 126.8 ng/mL, 3.2 ng/mL and 2087.0 ng/mL, respectively) and XBB (XMA01-mi3, XMA04-mi3 and XMA09-mi3 with IC_50_ values 48.9 ng/mL, 4.2 ng/mL and 4476.0 ng/mL, respectively) (Fig. 1d–f). The multivalent transformation also led to a 100-times increase in the neutralization titers of the antibodies against BA.1.1 and BA.2.12.1; notably, multivalent XMA04-mi3 showed significantly higher neutralization potency against BA.4/5 and XBB than the parental XMA04 (15,798 times and 1,627 times, respectively). These efficacies in neutralization led to profound reductions in the IC_50_ values for XMA04-mi3 (< 10 ng/mL against all Omicron sublineages). XMA09-mi3 also showed a moderately higher neutralizing activity (6–24-fold) against Omicron sublineages than its parental antibody. Additionally, the multivalent transformation of the Fab domain could rescue the loss in neutralization against SARS-CoV-2 variants (Supplementary Fig. 5a–d). Taken together, these data reveal that engineering nAbs into a multivalent form can increase their neutralization potency against Alpha, Beta, Gamma and Delta variants, as well as against the highly evasive Omicron BA.4/5 and XBB sublineages.

We speculated that the enhanced neutralization potency and breadth of mnAbs XMA01-mi3, XMA04-mi3 and XMA09-mi3 resulted from an elevated avidity (i.e., functional affinity): indeed, whereas the parental nAbs bound to the prototypical spike protein with affinity K_D_ values in a less than nanomolar range (Supplementary Fig. 6a–d and 7a, b), mnAbs XMA01-mi3 (616-fold) and XMA04-mi3 (12-fold) showed dramatically higher avidity in binding to the prototype spike protein; this may be due to a much faster binding rate or slower dissociation rate from the tethered antigen (Supplementary Fig. 7a, b). XMA09-mi3 showed no change in avidity as compared with the parental XMA09; this matches with its less potent neutralization efficacy as compared with the other transformed antibodies. For the BA.4/5 sublineage, which evaded neutralization by the parental nAbs, XMA01 and XMA04, we noted that XMA01-mi3 and XMA04-mi3 exhibited subnanomolar avidity, with potent and broad inhibition of sublineage BA.4/5 infection (Supplementary Fig. 7c, d). XMA09 remained in the picomolar K_D_ range of binding affinity following the multivalent transformation. Consistent with our previous report, XMA01 and XMA04, but not XMA09, provided potent neutralizing activities by efficiently inhibiting the prototypical RBD binding site of ACE2 receptor molecule (Supplementary Fig. 7e–g). Notably, the nanoparticle-based multivalent transformation of XMA09 allowed it to effectively block ACE2 from binding to the prototypical RBD, indicating that the mi3 modification enhanced the neutralization potency of XMA09 by promoting its blocking activity. The nanoparticle-based mnAbs induced higher response units than did their corresponding parental nAbs, indicating that XMA01-mi3, XMA04-mi3 and XMA09-mi3 can engage more spike proteins simultaneously, and that the nanoparticle-based mnAbs have a higher capability of inter-spike crosslinking (Supplementary Fig. 7h–j). Overall, these findings demonstrate the potent and broad neutralizing activity of nanoparticle-based multivalent nAbs against SARS-CoV-2 variants, with evidence for enhanced avidity binding, substantial steric hindrance with ACE2, and inter-spike crosslinking capabilities. We show that the multivalent transformation can enhance the interaction between the Fc region and the Fc receptors (FcγRI, FcγRIIIa and FcRn) (Supplementary Fig. 8a-d). Therefore, we next tested the natural killer-dependent antibody-mediated cell cytotoxicity (ADCC) function of the mnAbs as compared with the parental nAbs.^6^ As expected, the multivalent transformation promoted ADCC for all 3 antibodies by enhancing avidity binding to Fc receptors (Supplementary Fig. 8e, f). Notably, XMA01-mi3, XMA04-mi3 and XMA09-mi3 rescued ADCC function against the BA.4/5 spike-expressing cells, and this was achieved to a level similar as that against the prototypical strain spike-expressing cells (Supplementary Fig. 8g, h). These findings suggest that multivalent transformation can enhance the interaction between the Fc portion of IgGs and their corresponding receptors to improve the in vivo half-life and ADCC function of mnAbs XMA01-mi3, XMA04-mi3 and XMA09-mi3.

Rationally designed antibody cocktails targeting non-overlapping antigenic sites can provide an expanded neutralization spectrum and maintain protection against the activities of SARS-CoV-2 escape mutations.^7^ As such, a novel, enhanced antibody cocktail (XMA01-mi3, XMA04-mi3, and XMA09-mi3), hereafter referred to as the XMA cocktail, is likely to be important in the fight against COVID-19 (Supplementary Fig. 9a). In addition, we also constructed a Mosaic-mi3 molecule that possibly crosslinks more spikes than the parental nAbs in all possible scenarios (Supplementary Fig. 9a, b). The Mosaic-mi3 simultaneously displays XMA01, XMA04, and XMA09 on the mi3 particle (Supplementary Fig. 9c). The resistant BA.4/5 sublineage abolished the synergic neutralizing activity of the nAbs cocktail because of the complete loss of the individual neutralization potency of each antibody (Fig. 1g). The XMA cocktail, on the other hand, exhibited potent neutralization against SARS-CoV-2 variants Alpha, Beta, Gamma and Delta (IC_50_ values less than 3 ng/mL) as well as against Omicron sublineages (2.0 ng/mL and 16.1 ng/mL). Compared with the original nAb cocktail, the XMA cocktail showed about 10-fold increased neutralization potency against BA.1.1, BA.2, and BA.2.12.1; notably, more than 100-fold increased neutralization against BA.4/5 and XBB (Supplementary Fig. 9d, e). The Mosaic-mi3 molecule, also incorporating specificities of XMA01, XMA04, and XMA09, potently neutralized Omicron sublineages, of which BA.4/5 and XBB showed no escape (Supplementary Fig. 2d and 9f–i). However, compared with the Mosaic-mi3 molecule, the XMA cocktail still exhibited stronger neutralizing activity against the SARS-CoV-2-related variants (Fig. 1g and Supplementary Fig. 9f, g). Together, these data demonstrate that the XMA cocktail, composed of mnAbs XMA01-mi3, XMA04-mi3, and XMA09-mi3, can potently neutralize SARS-CoV-2-related variants including Omicron sublineages BA.4/5 and XBB, better that a mixture of parental nAbs or a Mosaic-mi3 molecule.

Overall, the multimerization platform based on the mi3 nanoparticle provides a tool for engineering nAbs into a multivalent format, thereby rescuing the complete loss of neutralization potency against SARS-CoV-2 variants, even the notorious BA.4/5 and XBB variants. The potential universality of this platform should enable the wide-ranging use of next-generation antibody therapeutics against other infectious diseases and their variants of concern.

## Supplementary information

Supplemental Information

## Data Availability

The data and materials used in the current study are available from the corresponding authors upon reasonable request.

## References

[1] Wang S (2022). Three SARS-CoV-2 antibodies provide broad and synergistic neutralization against variants of concern, including Omicron. Cell Rep..

[2] Cao Y (2023). Imprinted SARS-CoV-2 humoral immunity induces convergent Omicron RBD evolution. Nature.

[3] Bruun TUJ, Andersson AC, Draper SJ, Howarth M (2018). Engineering a rugged nanoscaffold to enhance plug-and-display vaccination. ACS Nano..

[4] Rahikainen R (2021). Overcoming symmetry mismatch in vaccine nanoassembly through spontaneous amidation. Angew. Chem. Int. Ed. Engl..

[5] Xiong H (2022). The neutralizing breadth of antibodies targeting diverse conserved epitopes between SARS-CoV and SARS-CoV-2. Proc Natl Acad Sci USA..

[6] Hong Y (2022). Cell-based reporter assays for measurements of antibody-mediated cellular cytotoxicity and phagocytosis against SARS-CoV-2 spike protein. J Virol Methods..

[7] Baum A (2020). Antibody cocktail to SARS-CoV-2 spike protein prevents rapid mutational escape seen with individual antibodies. Science..

