# Supplementary material for: Nanoparticle-based multivalent human antibodies offer potent and broad neutralization against Omicron sublineages
Source: Signal Transduct Target Ther. 2023 Aug 2;8:284. doi: 10.1038/s41392-023-01512-6 (PMC10393973; doi:10.1038/s41392-023-01512-6)
Supplement: Supplementary file 1 — Supplemental Information [file 41392_2023_1512_MOESM1_ESM.docx]

**Nanoparticle-based Multivalent Human Antibodies Offer Potent and Broad Neutralization against Omicron Sublineages**

Yizhen Wang ^1, 2, 4^, Feibo Song ^1, 2, 4^, Siling Wang ^1, 2, 4,^ *, Miaolin Lan ^1, 2, 4^, Tingdong Li ^1, 2, 4^, Huilin Guo ^1, 2^, Yali Zhang ^1, 2^, Shengxiang Ge ^1, 2,^ *, Zizheng Zheng ^1, 2,^ *, Ningshao Xia ^1, 2, 3^

^1^ State Key Laboratory of Molecular Vaccinology and Molecular Diagnostics; National Institute of Diagnostics and Vaccine Development in Infectious Diseases, School of Public Health, School of Life Sciences, Xiamen University, Xiamen 361102, China

^2^ Xiang An Biomedicine Laboratory, Xiamen 361102, China

^3^ Research Unit of Frontier Technology of Structural Vaccinology of Chinese Academy of Medical Sciences, Xiamen 361102, China

^4^ These authors contributed equally.

* Corresponding authors: [silingwang@xmu.edu.cn](mailto:silingwang@xmu.edu.cn) (S.W.), [sxge@xmu.edu.cn](mailto:sxge@xmu.edu.cn) (S.G.), [zhengzizheng@xmu.edu.cn](mailto:zhengzizheng@xmu.edu.cn) (Z.Z.)

**This PDF file includes:**

Materials and Methods

References

Supplementary Fig. 1-9

**Materials and Methods**

**Cell lines**

Cell lines used in this study were obtained from the ATCC (H1299, BHK21, 293T and Vero) or Thermo Fisher Scientific Inc. (ExpiCHO cells). All cell lines used in this study were routinely tested for mycoplasma and found to be mycoplasma-free.

**Protein expression and purification**

To prepare Ab and Ab-SpyTag, the heavy and light chains of Ab or Ab-SpyTag were cloned into pTT5-H and pTT5-K vectors (Youbio, VT2202), respectively. The Ab-SpyTag heavy chain contains the heavy chain sequence, a (GGGGS)_2_ spacer and SpyTag sequence.^1^ ExpiCHO cells (ThermoFisher) were transiently transfected to express Ab or Ab-SpyTag based on standard protocols.^2^ SpyCatcher-mi3 particles were expressed in *E.coli* BL21 (DE3) RIPL cells (Agilent) transformed with pET28a-SpyCatcher-mi3 plasmid with an N-terminal 6x-His tag, and then purified as follows. The bacteria were harvested by centrifugation, resuspended in 10 mL lysis buffer (25 mM Tris–HCl pH 8.5 with 300 mM NaCl, 0.1 mg/mL lysozyme, 1 mg/mL cOmplete mini EDTA-free protease inhibitor (Merck) and 1 mM phenylmethanesulfonyl fluoride (Sigma-Aldrich)), lysed by sonication on ice and centrifuged for 30 min at 16,900×*g* at 4°C. The supernatant was collected, filtered to remove the insoluble material through 0.45 µm and 0.22 µm filters (Sartorius) and then precipitated by saturated ammonium sulfate at 4°C for 1h while mixing at 100 rpm. Precipitated particles were pelleted by centrifugation at 30,000×*g* for 30 min at 4°C. The collected pellet was resuspended into TBS pH8.5 (25 mM Tris–HCl, 150 mM NaCl) and dialyzed overnight at 4°C against TBS pH8.5. Finally, the SpyCatcher-mi3 was further purified by size exclusion chromatography (SEC).

**Assembly and purification of Ab-mi3 or mosaic-mi3**

Ab-mi3 and mosaic-mi3 particles were assembled through the SpyCatcher-mi3 particles incubated with Ab-SpyTag (a single Ab or an equimolar mixture of three Abs) at 1:3 molar ratio overnight at 4°C in TBS pH8.5. The conjugated homotypic or mosaic Ab-mi3 particles were purified by SEC on a Superdex 200 10/300 column (GE Healthcare) equilibrated with TBS pH8.5 (25 mM Tris–HCl with 150mM NaCl) and analyzed by SDS-PAGE.

**Dynamic light scattering (DLS)**

Ab-mi3 was centrifuged for 30 min at 16,900×*g* at 4°C and 50 µL of the supernatant was transferred to the instrument's cuvette. DLS measurements were performed on NanoBrook Series (Brookhaven Instruments) using the manufacturer's recommended settings. Particle Solutions and GraphPad Prism 8.0 were used to analyze DLS data and make graphs.

**Negative-staining electron microscopy (EM)**

Nanoparticles mi3 and Ab-mi3 were loaded onto 200-mesh carbon-coated grids for 1 min (Quantifoil Micro Tools) and stained with 2% phosphotungstic acid (pH 6.4) for 30 s. Grids were imaged in a FEI Tecnai T12 transmission electron microscope at 120 kV using a Gatan US1000 CCD camera.

**Freeze-thaw stability assay**

The Ab-mi3 particles in TBS pH8.5 were frozen at -80°C for 30 min and thawed at 25°C for 15 min and were done four times. After the freeze-thaw, the Ab-mi3 particles were centrifuged (16,900×*g* for 30 min at 4°C) to remove insoluble impurities. The concentration and the capability of the Ab-mi3 binding to the wild-type S protein of SARS-CoV-2 were measured respectively after 0, 2 or 4 freeze-thaw cycles. The non-frozen Ab-mi3 was defined as 100% soluble.

**Pseudovirus neutralization assay**

A lentiviral pseudovirus system was used to identify the neutralizing potency of the antibodies before and after the engineering modification. The pseudoviruses (SARS-CoV, SARS-CoV-2 prototype strain, Alpha, Beta, Gamma, Delta, BA.1, BA.1.1, BA.2, BA.2.12.1, BA.2.75, BA.4/5 and XBB) were produced as described previously.^3^ Briefly, serially-diluted antibodies were pre-incubated with pseudovirus for 1h at 37°C. The antibody and pseudovirus mixtures were then added to a monolayer of H1299-ACE2hR cells in 96-well cell culture plates. The fluorescence images of cells were collected and analyzed by Opera Phenix high-content imaging system (PerkinElmer) after being cultured at 37°C for 48 hours. The IC_50_ values of antibodies were calculated using the 4-parameter logistic (4PL) regression in GraphPad Prism 8.0.

**Surface plasmon resonance (SPR)**

The binding affinity of antibodies to S proteins of various SARS-CoV-2 strains was tested by Biacore 8K system. The CM5 chips were immobilized with spike proteins (prototype, Omicron BA.4/5). The running buffer was DPBS (YuanPei). Experiments were carried out with serially-diluted antibodies and performed as single-cycle kinetics at 25°C. The equilibrium dissociation constant (K_D_) was analyzed by Biacore Insight Evaluation Software fitting to a 1:1 binding model.

Biacore 8K system was also used to confirm the presence of multiple antibody molecules on a single particle. The prototype spike protein was amine-coupled to the CM5 chip. Following the capture of a certain concentration of antibody by the CM5 chip, sufficient or excessive amounts of prototype RBD protein were loaded onto the chip at 25°C.The SPR signal was fit to a 1:1 binding model by Biacore Insight Evaluation Software.

**ELISA binding assays**

The binding of Ab-SpyTag to SARS-CoV-2 spike protein was analyzed by ELISA as described previously^2^. Briefly, the antibodies were diluted according to a concentration gradient and added to the microwell plates coated with S protein (100 ng per well). After incubating for 30 min at 37°C, the microwell plates were washed five times with PBST. Then, 100 μL HRP-conjugated goat anti-human IgG antibody at 1:5000 (Abcam) was added and incubated for 30 minutes at 37 °C. The microplates were washed five times with PBST again. Finally, 100 µL of tetramethylbenzidine chromogen (TMB) solution (Wantai BioPharm) was incubated for 15 minutes at 37 °C. The reaction was stopped by 50 µL of 2M H_2_SO_4_. The microwell plate was read at 450 nm with a baseline wavelength of 630 nm. The EC_50_ values of the antibodies were calculated using the 4-parameter logistic (4PL) regression in GraphPad Prism 8.0.

**The competing binding assay of antibodies with ACE2**

Antibodies at the initial concentration of 100 µg/mL were 4-fold gradient diluted. The serially-diluted antibodies were added to 96-well microplates coated with SARS-CoV-2 spike protein and incubated at 37°C for 30 min. 1 × DPBS (YuanPei) served as a control. Then HRP-conjugated ACE2-hFc (Wantai BioPharm) was added at a certain concentration (OD values 1~2) and incubated again at 37°C for 30 min. Then the microplates were washed five times with PBST, incubated with TMB solution (Wantai BioPharm) and terminated with 2M H_2_SO_4_. At 450 nm wavelength, the microwell plates were read, with a baseline wavelength of 630 nm. The blocking rate of antibodies was calculated as follows: [1-(OD_Ab_/OD_PBS_)] X 100%. The blocking IC_50_ of antibodies was calculated using the 4-parameter logistic (4PL) regression in GraphPad Prism 9.0.

**Antibody-dependent cellular cytotoxicity function assay**

Antibody-dependent cellular cytotoxicity (ADCC) was performed as previously described.^4^ In brief, 20 µL of the serially-diluted antibodies were added to the SFLwt or SFLfk target cells and incubated for 1 h at 37°C in a 5% CO_2_ incubator. Jurkat-NFAT-hCD16 effector cells were then added to the mixture of antibody and target cells and cultured at 37°C for 18 h in a 5% CO_2_ incubator. Finally, the bioluminescence intensity of the cell supernatant was detected by a multimode microplate reader (Spark 20 M, Tecan).

**Quantification and statistical analysis**

GraphPad Prism (version 8.0.1) was used for all statistical calculations. IC_50_ values were calculated by non-linear regression analysis (log(agonist) vs response - Variable slope (four parameters)).

**References:**

1 Rahikainen, R. *et al.* Overcoming Symmetry Mismatch in Vaccine Nanoassembly through Spontaneous Amidation. *Angew Chem Int Ed Engl*. **60**, 321-330, (2021).

2 Wang, S. *et al.* Three SARS-CoV-2 antibodies provide broad and synergistic neutralization against variants of concern, including Omicron. *Cell Rep*. **39**, 110862, (2022).

3 Zhang, Y. *et al.* Cross-species tropism and antigenic landscapes of circulating SARS-CoV-2 variants. *Cell Rep*. **38**, 110558, (2022).

4 Hong, Y. *et al.* Cell-based reporter assays for measurements of antibody-mediated cellular cytotoxicity and phagocytosis against SARS-CoV-2 spike protein. *J Virol Methods*. **307**, 114564, (2022).

**
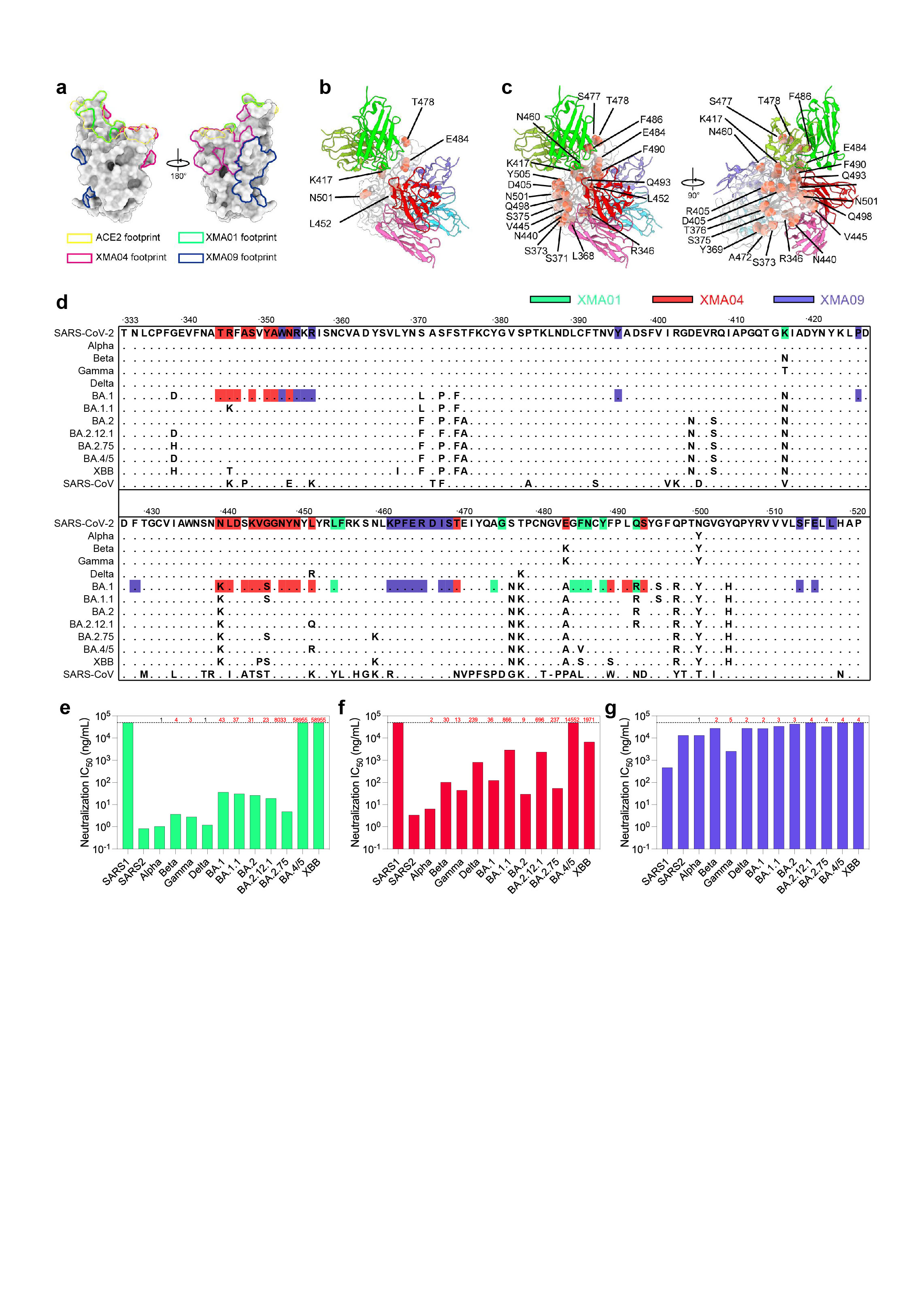
**

**Supplementary Fig. 1.** **Complete loss of neutralization potency of nAbs XMA01, XMA04 and XMA09 against Omicron sublineages. a** Comparisons of the antigenic sites among XMA01 (spring green line), XMA04 (crimson line), and XMA09 (dodger blue line) on prototype-RBD (gray surface representation) (PDB: 7WHZ). The footprint of ACE2 is marked as the yellow line. **b-c** Structure of prototype RBD and XMA01/XMA04/XMA09 with highlighted mutations in the Alpha, Beta, Gamma and Delta variants (**b**) and Omicron sublineages BA.1, BA.1.1, BA.2, BA.2.12.1, BA.2.75, BA.4/5 and XBB (**c**) (displayed in coral sphere). **d** Sequence alignment of the spike proteins of SARS-CoV-2, VOCs, Omicron sublineages and SARS-CoV with strictly conserved residues shown as dots, and the antigenic sites of XMA01, XMA04 and XMA09 are indicated in green, red and purple, respectively. **e-g** Neutralization potency of nAbs XMA01 (**e**), XMA04 (**f**) and XMA09 (**g**) against the SARS-CoV (SARS1), SARS-CoV-2 prototype (SARS2), VOCs and Omicron sublineages. The IC50 values were determined using LV pseudovirus with SARS-CoV-2 spike protein. The fold changes of neutralization potency against VOCs and Omicron sublineages related to SARS2 are presented in the panel, and the limit of detection is 50 µg/mL (dash line). The experiments in (**e-g**) were repeated in triplicate.

**
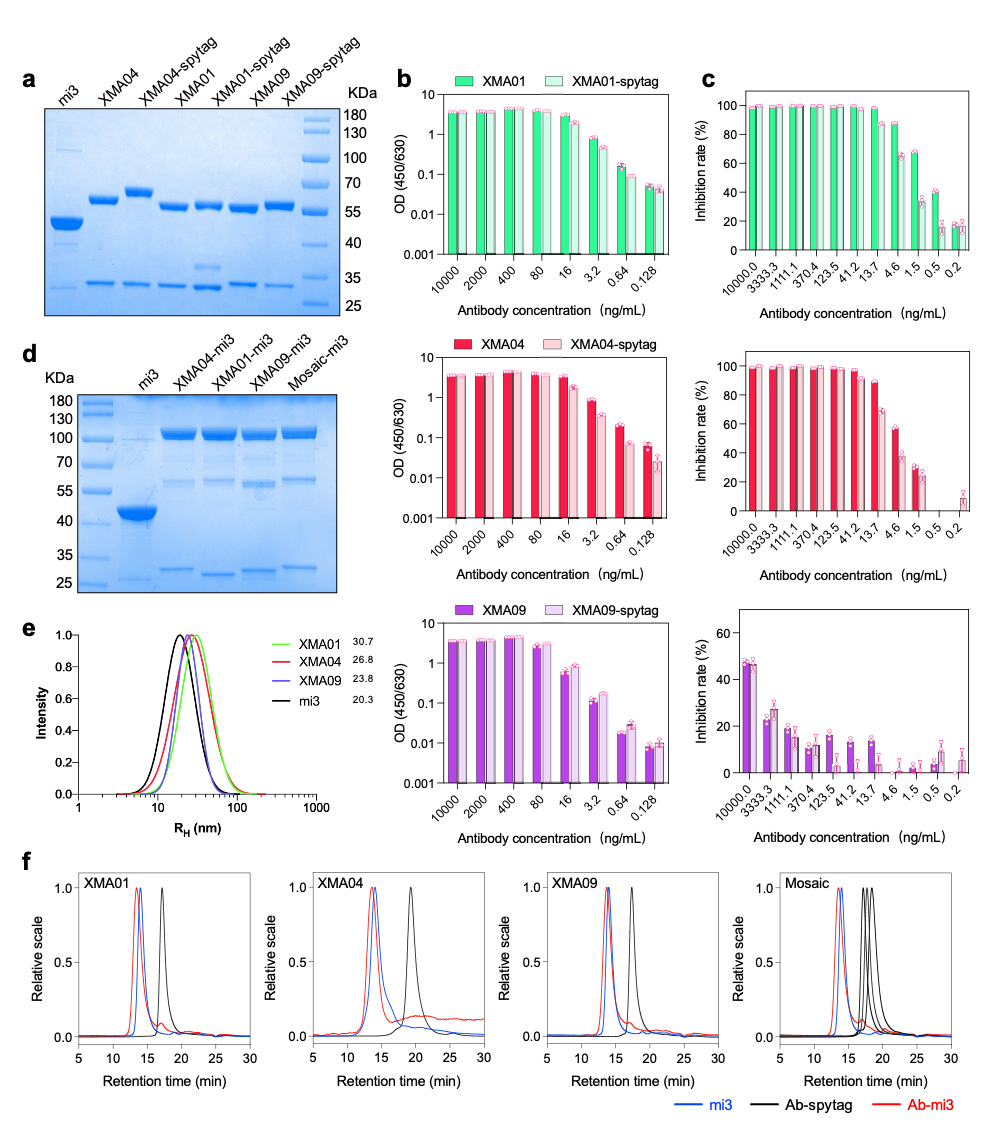
**

**Supplementary Fig. 2 Nanoparticle mi3-based multivalent transform of anti-SARS-CoV-2 nAbs. a** SDS-PAGE of nanoparticle mi3, Ab (XMA01, XMA04 and XMA09) and Ab-SpyTag (XMA01-SpyTag, XMA04-SpyTag and XMA09-SpyTag). **b** Binding activity of Ab and Ab-SpyTag to the spike protein of SARS-CoV-2 prototype strain. The experiments were repeated in triplicate. Data are expressed by means ± s.d. (n=3). **c** Neutralizing activity of Ab and Ab-SpyTag against LV pseudovirus of SARS-CoV-2 prototype strain. The experiments were repeated in triplicate. Data are expressed by means ± s.d. (n=3). **d** SDS-PAGE of nanoparticle mi3 and Ab-mi3 (mnAbs: XMA01-mi3, XMA04-mi3 and XMA09-mi3). **e** Hydrodynamic radius (R_H_) of the nanoparticle mi3 and Ab-mi3 (mnAbs: XMA01-mi3, XMA04-mi3 and XMA09-mi3). **f** Size exclusion chromatography of nanoparticle mi3, Ab-SpyTag (XMA01-SpyTag, XMA04-SpyTag and XMA09-SpyTag), Ab-mi3 (mnAbs: XMA01-mi3, XMA04-mi3 and XMA09-mi3) and Mosaic-mi3 molecular.

**
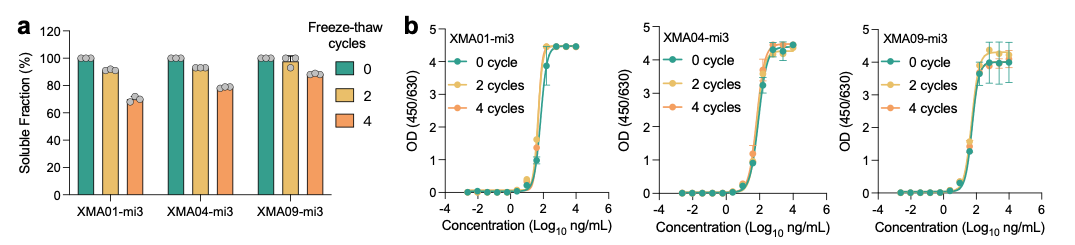
**

**Supplementary Fig. 3 Stability of nanoparticle mi3-based multivalent nAbs. a** mnAbs remained soluble after freeze−thaw and soluble components were quantified. **b** The binding activities of soluble mnAbs to prototype strain spike protein were tested by ELISA. Data are expressed by means ± s.d. (n=3).


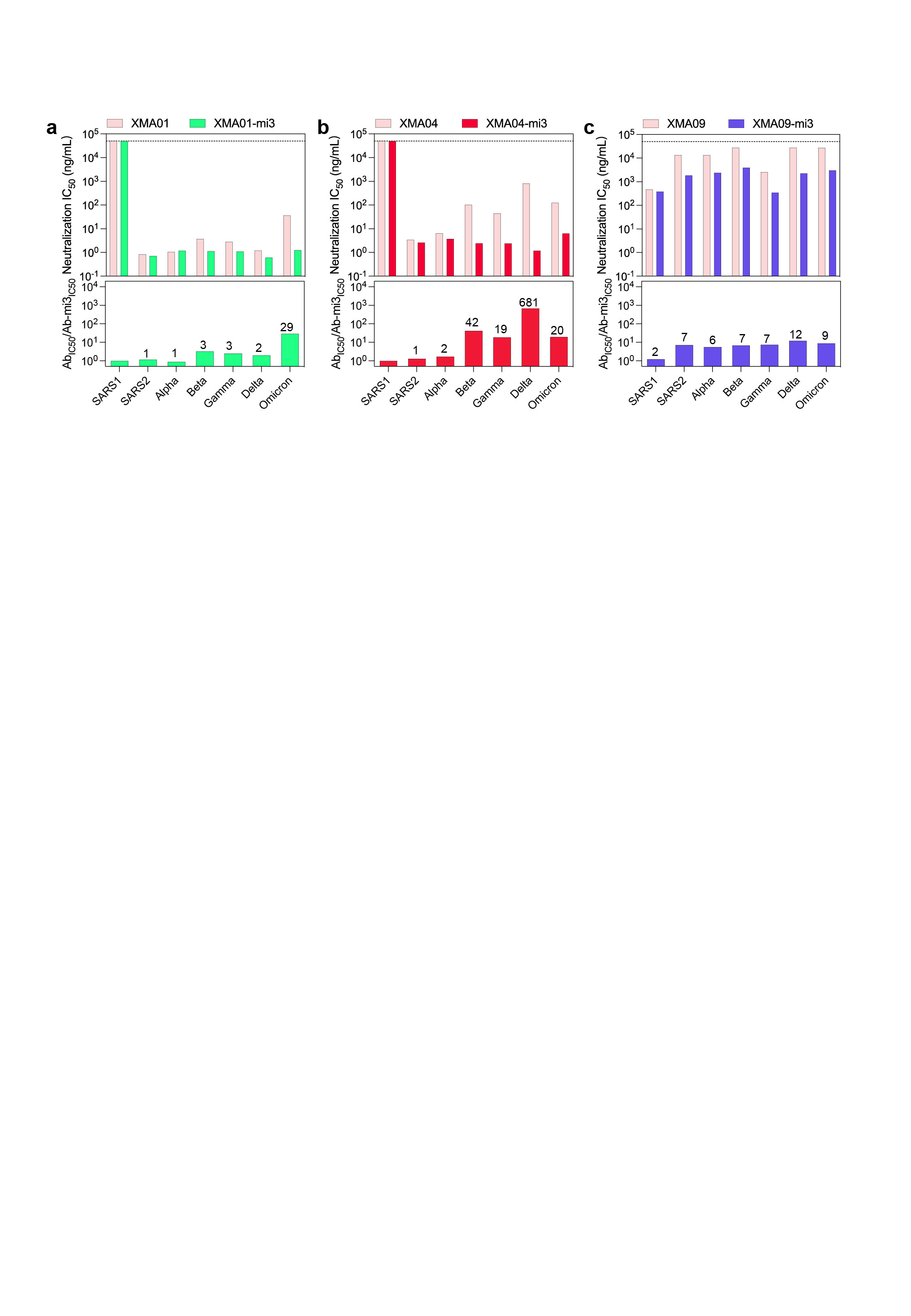


**Supplementary Fig. 4** Neutralization potency of XMA01/XMA01-mi3 (**a**), XMA04/XMA04-mi3 (**b**) and XMA09/XMA09-mi3 (**c**) against the SARS-CoV (SARS1), SARS-CoV-2 prototype (SARS2) and VOCs.


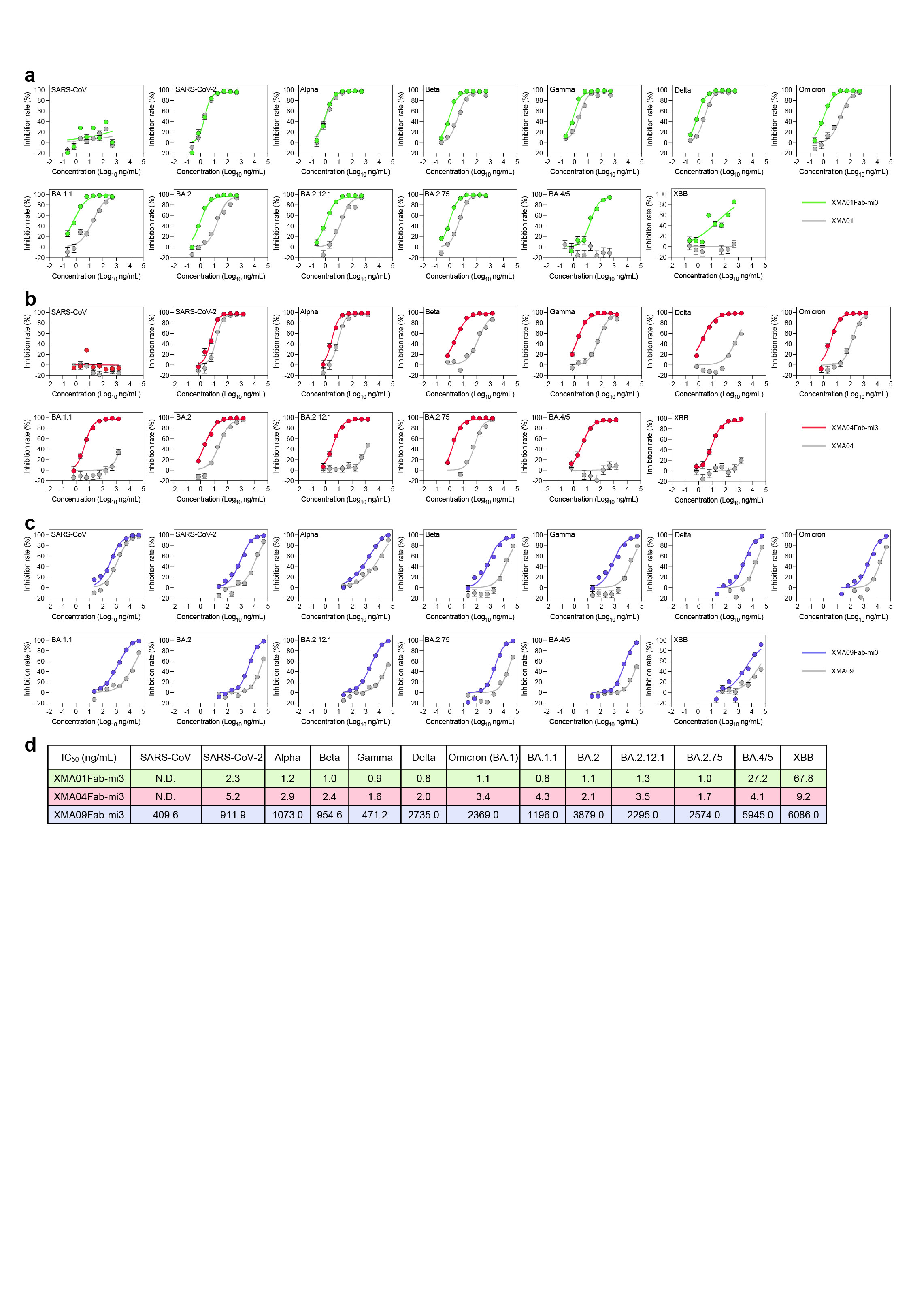


**Supplementary Fig. 5 Enhanced neutralization potency of nanoparticle mi3-based multivalent nAb-Fabs. a-c** The neutralization potency of nanoparticle mi3-based multivalent nAb-Fabs, including XMA01Fab-mi3 (**a**), XMA04Fab-mi3 (**b**) and XMA09Fab-mi3 (**c**), were tested using LV pseudovirus with spike proteins of SARS-CoV, SARS-CoV-2 prototype, VOCs and Omicron sublineages, with XMA01, XMA04 and XMA09 as controls. The experiments were repeated in triplicate. Data are expressed by means ± s.d. (n=3). **d** The IC_50_ values of XMA01Fab-mi3, XMA04Fab-mi3 and XMA09Fab-mi3 were displayed.


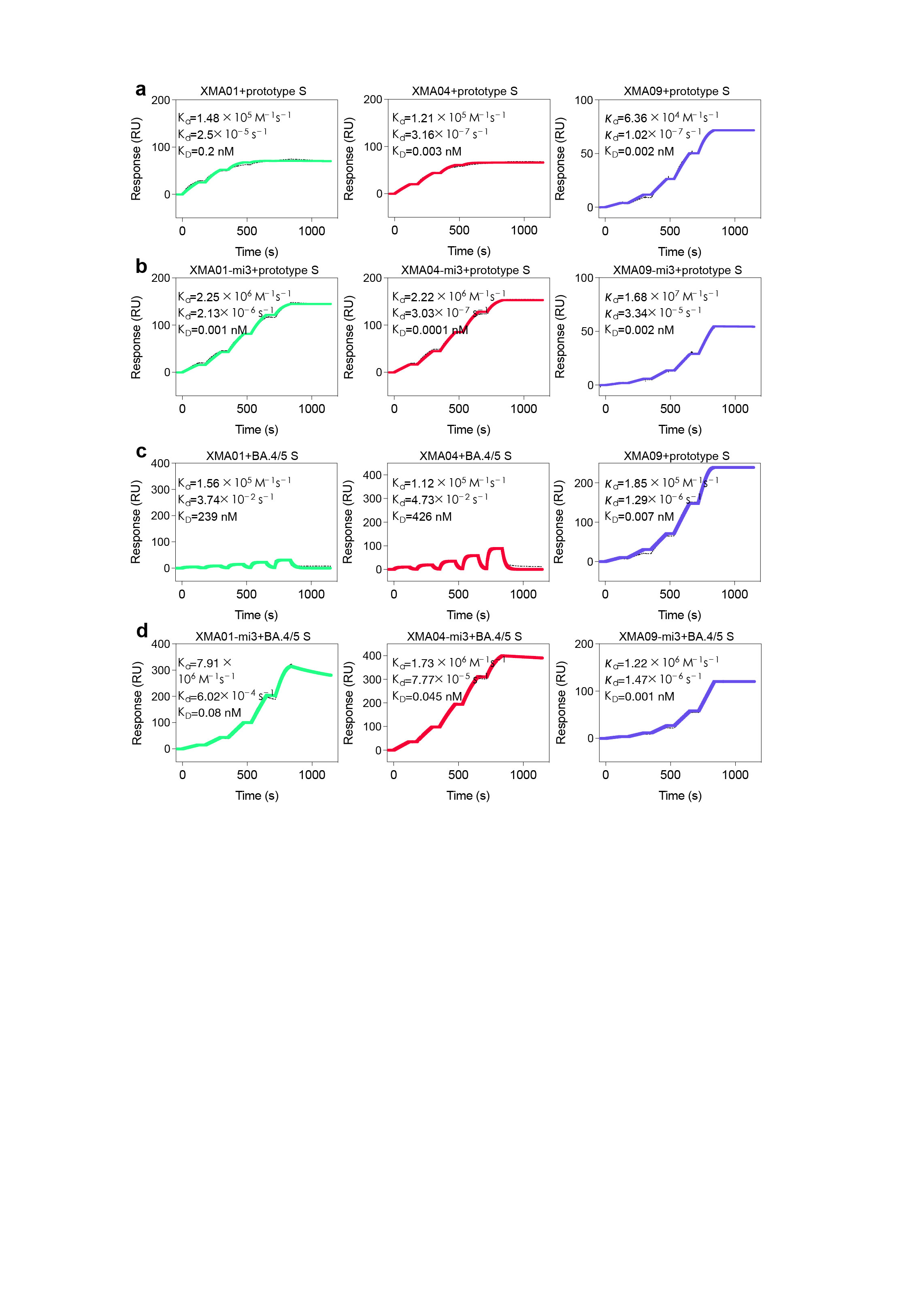


**Supplementary Fig. 6** Representative SPR traces indicating the avidity binding of XMA01/XMA01-mi3 (left), XMA04/XMA04-mi3 (center) and XMA09/XMA09-mi3 (right) to prototype strain spike (**a-b**) and BA.4/5 spike (**c-d**) immobilized at 150 nM on the SPR chip. The green (XMA01/XMA01-mi3), red (XMA04/XMA04-mi3) and purple (XMA09/XMA09-mi3) bold lines represent raw data, whereas dash lines represent global fits. The experiments were repeated independently at least triple (n=3) and one representative result was shown.

**
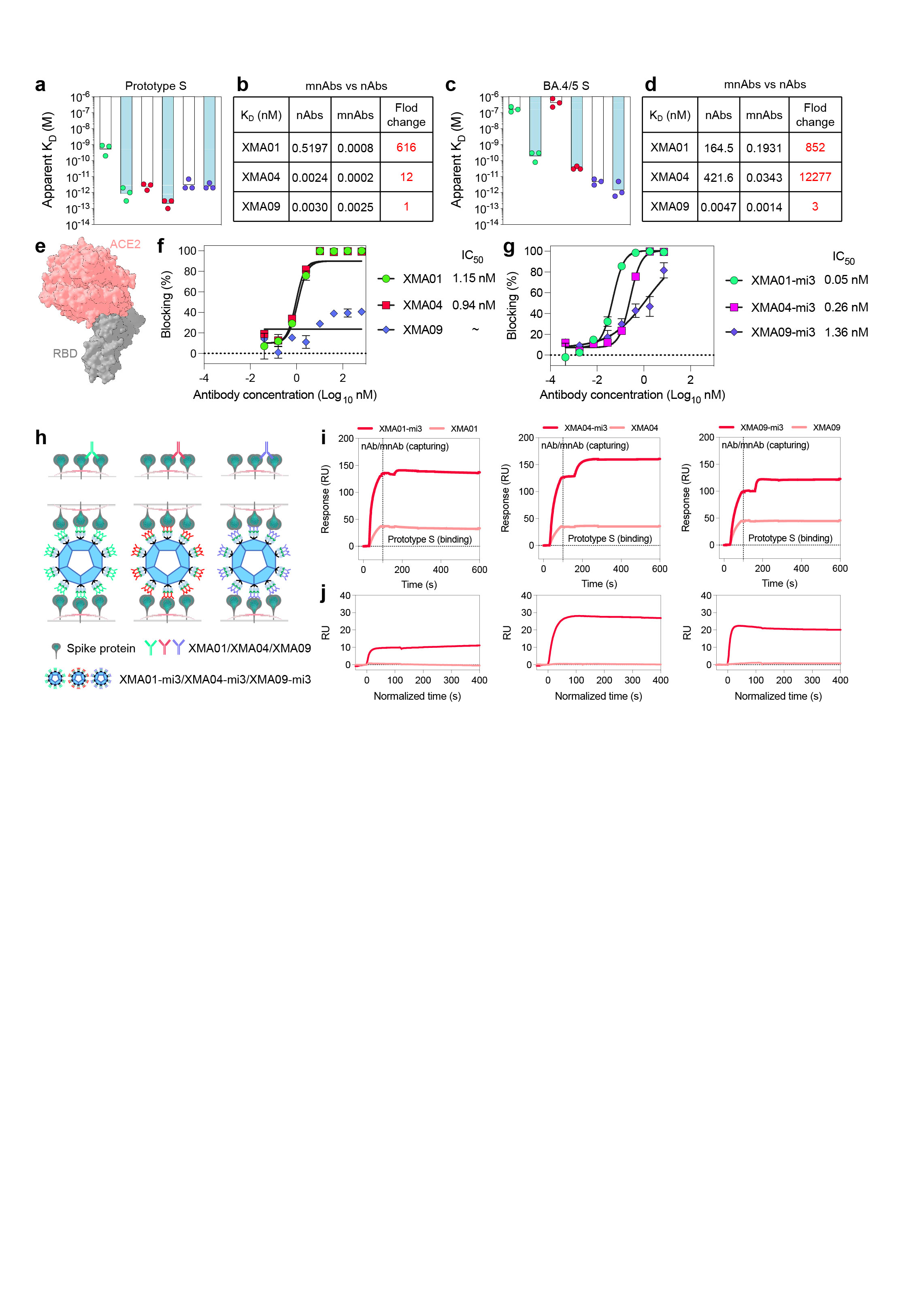
**

**Supplementary Fig. 7 Mechanisms of enhanced neutralization potency of mnAbs. a-d** Avidity binding (apparent K_D_) of nAbs (XMA01, XMA04 and XMA09) and mnAbs (XMA01-mi3, XMA04-mi3 and XMA09-mi3) to the prototype strain spike (**a-b**) and BA.4/5 spike (**c-d**). The K_D_ values in (**a**,**c**) are expressed as the means ± s.d. (n=3), fold change of K_D_ means of mnAbs vs nAbs are shown in (**b**, **d**). **e** Domain-colored maps of the structure of RBD and receptor ACE2. **f-g** Blocking activity of nAbs (XMA01, XMA04 and XMA09) (**f**) and mnAbs (XMA01-mi3, XMA04-mi3 and XMA09-mi3) (**g**) against ACE2 binding to RBD, shown as the percentage of blocked ACE2 at different concentrations of antibodies. The experiment was repeated in duplicate. Data are expressed by means (n=2). **h** A diagram showing the inter-spike crosslinking activity of nAbs (XMA01, XMA04 and XMA09) and mnAbs (XMA01-mi3, XMA04-mi3 and XMA09-mi3). **i-j** Multivalent binding to spike by nAbs and mnAbs. (**i**) The sensorgrams showing the capturing of indicated nAbs and mnAbs by immobilized spike protein and the following binding by free spike protein. (**j**) The binding to free spike protein is normalized. The experiments in (**i-j**) were repeated independently at least twice and one representative result is shown.

**
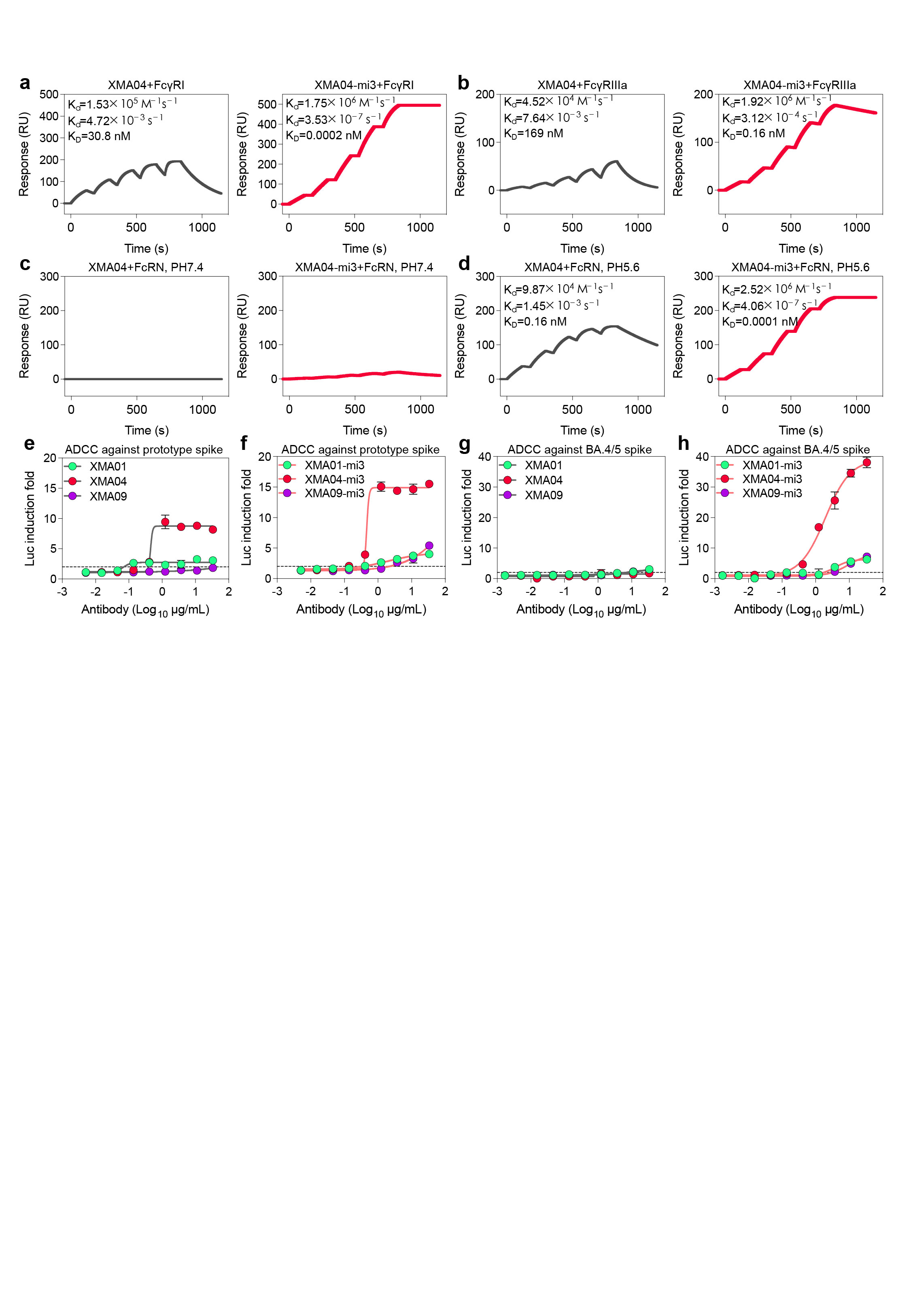
**

**Supplementary Fig. 8 Enhanced affinity between multivalent nAbs and Fc receptors. a-d** The enhanced affinity of mnAbs binding to Fc receptors. Sensograms of nAbs and mnAbs binding to FcγRI (**a**), FcγRIIIa (**b**), FcRn at physiological pH (**c**) and FcRn at endosomal pH (**d**). Bold gray lines and bold red lines represent raw data of nAbs and mnAbs, respectively, whereas dash lines represent global fits. The experiments in (**a-d**) were repeated independently at least twice and one representative result was shown. **e-h** ADCC function of nAbs and mnAbs against prototype strain (**e-f**) and BA.4/5 variant (**g-h**). Data are expressed by means ± s.d. (n=3).

**
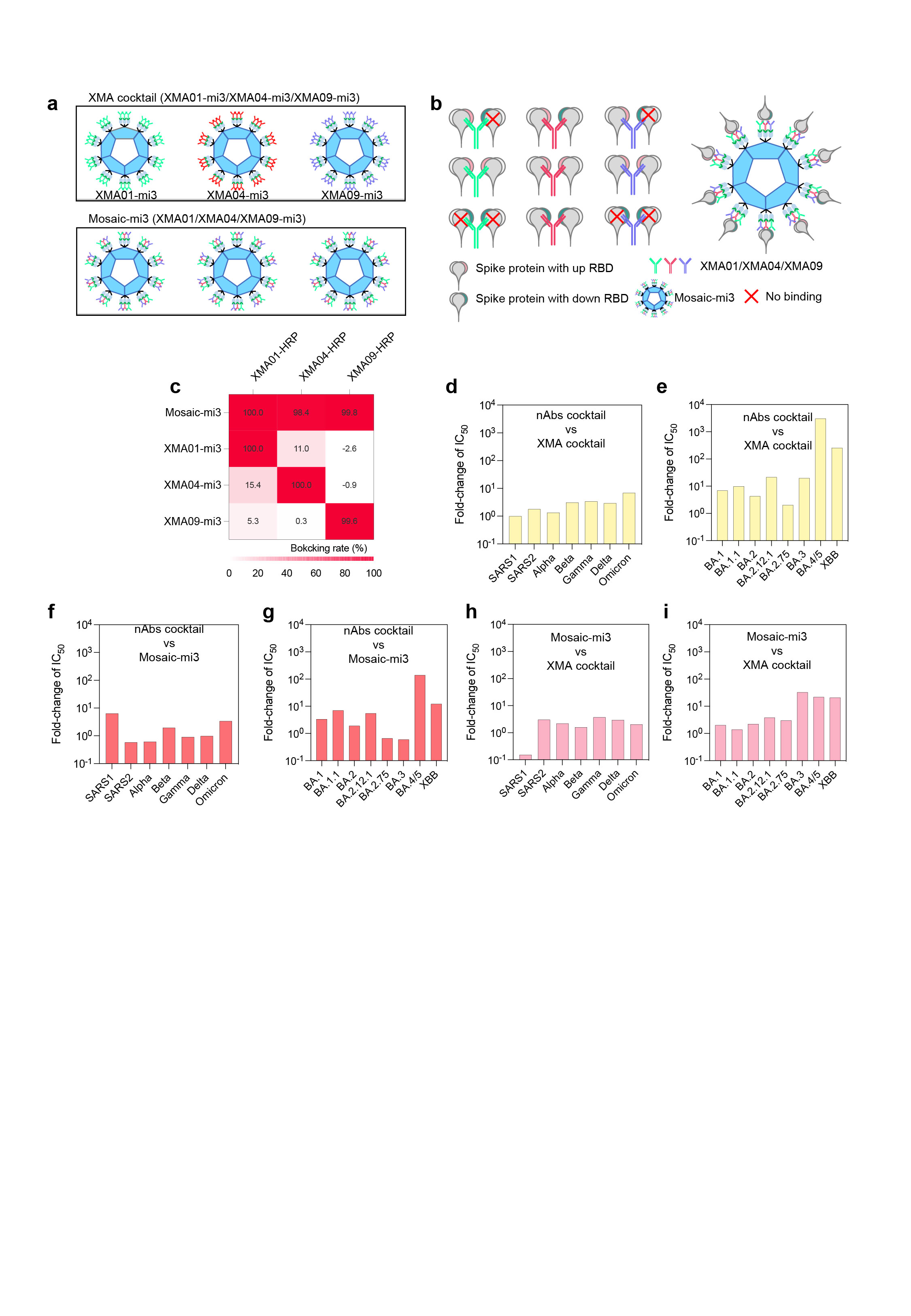
**

**Supplementary Fig. 9 Multivalent nAbs cocktail showing broad and potent neutralizing activity. a** Schematic representation of XMA cocktail and Mosaic-mi3. **b** The enhanced binding activity of Mosaic-mi3 to spikes with diverse state. **c** Representative blocking assay indicating the blocking activity of Mosaic-mi3 molecular against HRP-conjugated XMA01, XMA4 and XMA09 binding to the mobilized spike protein, with XMA01-mi3, XMA04-mi3 and XMA09-mi3 as controls. **d-e** Fold change of IC_50_ values of nAbs cocktail against SARS-CoV, SARS-CoV-2 prototype strain, VOCs and Omicron sublineages, compared to that of XMA cocktail. **f-g** Fold change of IC_50_ values of nAbs cocktail against SARS-CoV, SARS-CoV-2 prototype strain, VOCs (**f**) and Omicron sublineages (**g**), compared to that of Mosaic-mi3 molecular. **h-i** Fold change of IC_50_ values of Mosaic-mi3 against SARS-CoV, SARS-CoV-2 prototype strain, VOCs and Omicron sublineages, compared to that of XMA cocktail.
